# Infection Counter: Automated Quantification of in Vitro Virus Replication by Fluorescence Microscopy

**DOI:** 10.3390/v8070201

**Published:** 2016-07-21

**Authors:** Siân Culley, Greg J. Towers, David L. Selwood, Ricardo Henriques, Joe Grove

**Affiliations:** 1 MRC Laboratory for Molecular Cell Biology, University College London, London WC1E 6BT, UK; s.culley@ucl.ac.uk (S.C.); r.henriques@ucl.ac.uk (R.H.); 2 Department of Cell and Developmental Biology, University College London, London WC1E 6BT, UK; 3 Division of Infection and Immunity, University College London, London WC1E 6BT, UK; g.towers@ucl.ac.uk; 4 Wolfson Institute for Biomedical Research, University College London, London WC1E 6BT, UK; d.selwood@ucl.ac.uk

**Keywords:** hepatitis C virus, ImageJ, image quantification, plaque assay, focus-forming assay

## Abstract

The ability to accurately and reliably quantify viral infection is essential to basic and translational virology research. Here, we describe a simple and robust automated method for using fluorescence microscopy to estimate the proportion of virally infected cells in a monolayer. We provide details of the automated analysis workflow along with a freely available open-source ImageJ plugin, Infection Counter, for performing image quantification. Using hepatitis C virus (HCV) as an example, we have experimentally verified our method, demonstrating that it is equivalent, if not better, than the established focus-forming assay. Finally, we used Infection Counter to assess the anti-HCV activity of SMBz-CsA, a non-immunosuppressive cyclosporine analogue.

## 1. Introduction

Robust and reliable infectivity assays are an essential part of a virologist’s tool kit. The classical plaque assay provides a tried and tested method of determining virus titer; however, it can only be applied to cytopathic viruses (plaques arise through virus-mediated cell death within a monolayer) and typically requires plaques to grow until visible to the naked eye. A related technique, the focus-forming assay (FFA), can be used to titer non-cytopathic viruses. This relies on the detection of infected cells by immunostaining for viral antigen or via a genetically encoded fluorescent reporter. However, the FFA typically requires manual identification and counting of foci by fluorescence microscopy; this is extremely time consuming, vulnerable to human error and has a limited linear dynamic range.

The FFA is a mainstay of hepatitis C virus (HCV) basic research but the limitations of this method constrain experimental throughput. As a consequence, various groups have developed in-house automated quantification methods [1,2,3]; however, these generally require bespoke equipment and/or proprietary software, and often possess the same limited dynamic range of the FFA. In this report we describe Infection Counter, a method for automated quantification of in vitro HCV replication using a simple and yet robust data analysis pipeline to estimate the percentage of infected cells. When combined with a plate-reading fluorescence microscope, this provides a medium/high-throughput assay for basic and translational research. We validate our method using a known inhibitor of HCV replication and then use Infection Counter to assess the inhibitory activity of a cyclosporine analogue. Infection Counter is provided as an easy-to-use open-source plugin for the popular ImageJ or Fiji image analysis software [4,5] that can be adapted to study a wide range of other viruses.

## 2. Materials and Methods

### 2.1. Cell Lines

Huh-7.5 cells (provided by APATH LLC) were propagated in Dulbecco’s Modified Eagle Medium (DMEM) + 10% fetal calf serum (FCS) supplemented with penicillin, streptomycin and non-essential amino acids (all from Life Technologies, Carlsbad, CA, USA).

### 2.2. Antibodies

Mouse anti-NS5A monoclonal antibody (mAb) 9E10 was a gift from Charles M. Rice, Rockefeller University, NY, USA. Mouse anti-CD81 mAb 2.20 was a gift from Jane A. McKeating, University of Birmingham, UK.

### 2.3. Generation and Propagation of Cell-Culture-Proficient HCV (HCVcc)

Full-length HCVcc RNA genomes were generated by in vitro transcription from *Xba*I linearized J6/JFH plasmid template (provided by APATH LLC). To initiate infection, viral RNA was introduced in to Huh-7.5 cells by electroporation using a BTX830 (Harvard Instruments, Cambridge, UK) [6]. High-titer cell-culture-adapted HCVcc was generated by continuous culture in Huh-7.5 cells for 20 weeks; thereafter, HCVcc stocks were generated by harvesting secreted virus in serum-free DMEM every 2 h throughout the day for 3 days.

### 2.4. Infectivity Assay

Huh-7.5 cells were seeded at a density of 1.5 × 10^4^ in to each well of a standard flat bottomed 96-well tissue culture test plate 24 h prior to study. On the day of infection, the cell culture media in each well was replaced with 50 μL DMEM + 6% FCS and inoculated with 50 μL of virus (diluted appropriately in serum-free DMEM). After 48 h the cells were washed once in 50 μL PBS and fixed with 250 μL ice-cold methanol for 10 min. To detect viral antigen, samples were blocked with 2% bovine serum albumin (BSA) + 0.1% Triton-X 100 and stained with 100 ng/mL mouse anti-NS5A 9E10, followed by 2 μg/mL goat anti-mouse Alexa Fluor 488 secondary antibody (Life Technologies). Nuclear DNA was counterstained with 2 μg/mL 4′,6-diamidino-2-phenylindole (DAPI) for 20 min.

### 2.5. Focus-Forming Assay

Infectivity assays were performed with a serial dilution of HCVcc. Discrete foci of HCV positive cells were manually counted in replicate wells across a range of virus dilutions. For each dilution the titer was calculated by multiplying the mean number of foci per well by the dilution factor and a volume correction, resulting in a value expressed in focus-forming units per mL (FFU/mL). The final titer was derived by taking the average of three dilutions from across the series.

### 2.6. Microscopy

Fixed and stained samples in standard 96-well plates were imaged using a Nikon Ti inverted microscope fitted with a motorized encoded stage for plate-reading. A 3.5 mm by 3.5 mm area of each well was acquired by image stitching using an ORCA Flash 4 sCMOS camera (Hamamatsu, Welwyn Garden City, UK), with 405 nm and 488 nm fluorescence illumination provided by a PE4000 LED (CoolLED, Andover, UK) unit through a multi-band excitation/emission filter cube (Semrock, Rochester, NY, US). To ensure optimal imaging, software-based autofocusing was performed prior to acquiring each well. The images were exported from NIS elements as 8 bit tif files for analysis in Fiji.

### 2.7. Inhibition Experiments

For receptor blockade, Huh-7.5 cells were pre-treated with 50 μL/well anti-CD81 2.20, diluted in DMEM + 6% FCS, for 1 h at 37 °C, after which they were inoculated with 50 μL of virus appropriately diluted in serum free DMEM. For SMBz-CsA treatment, Huh-7.5 cells were pretreated with 100 μL/well of SMBz-CsA, diluted in DMEM + 3% FCS, for 1 h at 37 °C, after which they were inoculated with 10 μL of appropriately diluted virus, the inoculum was removed after 6 h and the cells re-fed with DMEM + 3% FCS without drug. In both cases samples were prepared as described in the infectivity assay, above.

### 2.8. Statistical Analysis

Statistical analysis and curve fitting was performed using Prism 6 (GraphPad, La Jolla, CA, USA). Fit models and statistical tests used are indicated in the text and figure legends.

## 3. Results

### 3.1. Development of Infection Counter

**Figure 1:**
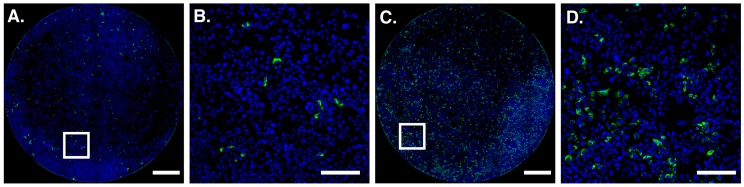
Huh-7.5 human hepatoma cells infected with J6/JFH HCVcc. Cells were fixed 48 h post-inoculation and stained for viral antigen NS5A (green), nuclear DNA was counterstained with 4′,6-diamidino-2-phenylindole (DAPI) (blue). (**A**) and (**C**) display representative wells with sparse and dense infection, respectively; (**B**) and (**D**) are enlargements of the highlighted areas. Scale bars 1000 μm (**A**) and (**C**) 200 μm (**B**) and (**D**).

In a typical HCV in vitro infectivity assay, human hepatoma cells are inoculated with HCVcc for a defined time period during which virus particles bind to cells, undergo entry and initiate infection. Experimental observations indicate that HCV protein synthesis and genome replication is detectable within 10–24 h and de novo virion production is apparent by 18–48 h [6,7,8]. Therefore, it is possible for 1–2 rounds of replication to occur within 48 h of inoculation. Consequently, at this time point HCVcc infection manifests as small foci typically containing 1–8 infected cells (Figure 1A,B); these arise both through limited spread of the virus and division of infected cells. When sufficiently sparse, individual foci are easily distinguished allowing manual counting and determination of virus titer. However, this process is labor intensive and vulnerable to user error and bias. Moreover, when spatially crowded, individual foci cannot be discerned (Figure 1C,D); this limits the linear dynamic range of the assay to a maximum of ~150 foci per well of a 96-well plate.

**Figure 2:**
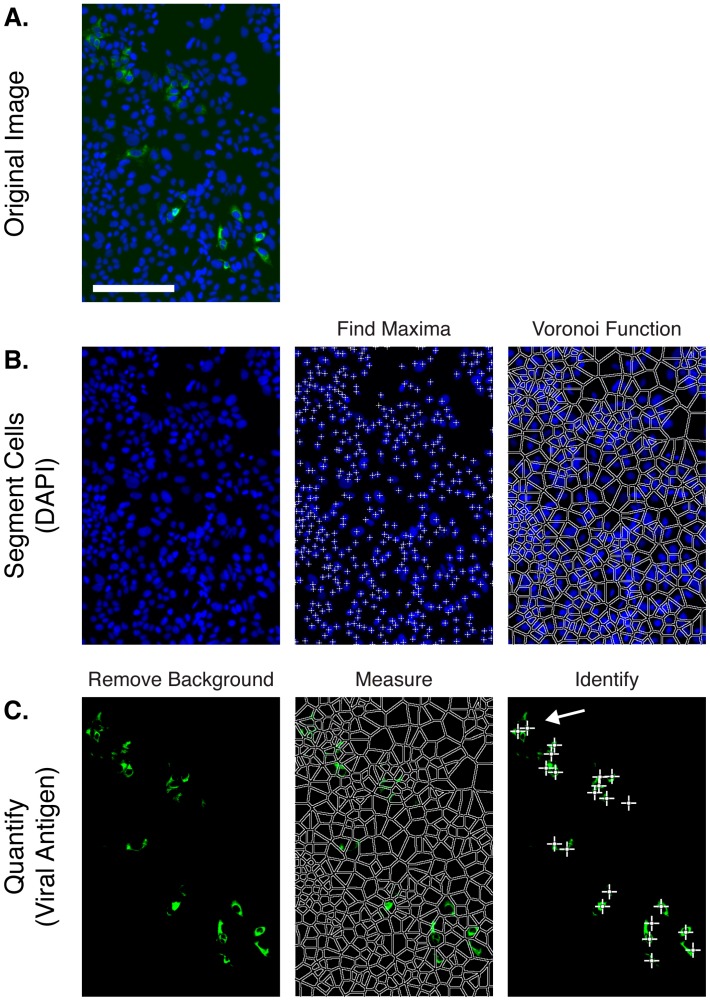
Infection Counter analysis workflow. (**A**) A representative unprocessed image of Huh-7.5 cells infected with J6/JFH HCVcc, cells were fixed 48 h post-inoculation and stained for viral antigen NS5A (green), nuclear DNA was counterstained with DAPI (blue), scale bar 200 μm; (**B**) Cells were first segmented using the DAPI channel: individual nuclei were identified using the in-built ImageJ ‘Find Maxima’ function, and the locations of these were then used to generate a Voronoi mosaic (in-built ImageJ ’Voronoi’ function) to approximate individual cell bodies; (**C**) Following background correction, the fluorescence intensity of viral antigen associated with each cell was measured and positive cells were identified using an empirically chosen signal threshold (in this image 4.86% of cells were scored as positive). The analysis method correctly identified the majority of cell culture proficient (HCVcc) infected cells (white crosshairs), but miscounted a minority of cells; for instance, the white arrow indicates a group of four cells that has been scored as two by the algorithm.

Our aim was to build an automated image analysis pipeline to estimate the percentage of infected cells in fluorescence microscopy images such as those shown in Figure 1. We devised the ImageJ plugin Infection Counter, a simple and yet robust analytical process that segments cells based on DAPI-stained nuclei and then estimates the proportion of positive cells based on thresholding of associated viral antigen fluorescence signal. A summary of the Infection Counter workflow is shown in Figure 2. We provide the software to perform this analysis in the form of an ImageJ plugin along with detailed instructions of how to use the plugin (Supplementary Note 1), alternatively the latest version and source code of the plugin can be found in the InfectionCounter GitHub repository [9].

### 3.2. Validation

**Figure 3:**
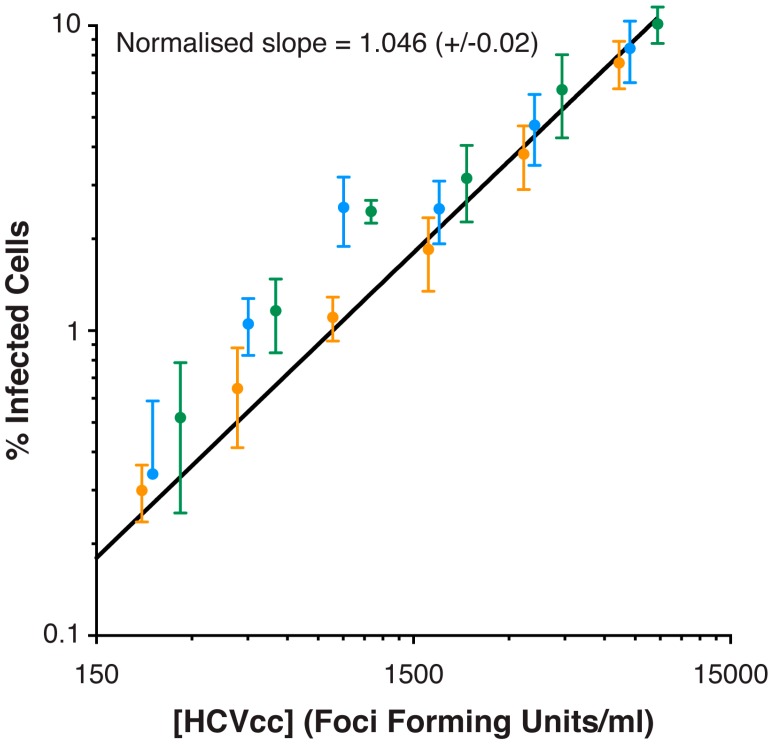
Serial dilution of HCVcc. Three independent stocks of J6/JFH HCVcc (green, blue and orange markers) were serially diluted from 1/400 to 1/102400 and inoculated on to Huh-7.5 cells in replicates of six. The cells were fixed at 48 h, stained for viral antigen and nuclear DNA, and imaged using a plate-reading fluorescence microscope. The titer of each stock was calculated using the standard FFA (as described in materials and methods) and the proportion of infected cells at each concentration was estimated using Infection Counter. The plot displays concentration of HCVcc (extrapolated from the viral titer) versus the estimated percentage of infected cells. Linear regression was performed and produced a line of best of fit which, when normalized for differences in units, exhibited a slope of 1.046, indicating a near-perfect linear relationship between the concentration of HCVcc and estimated proportion of infected cells. Error bars indicate standard deviation from the mean, *n* = 6.

Visual inspection of the processed images suggested that Infection Counter was performing a good job of identifying infected cells (Figure 2). In cases where infected cells are tightly clustered, Infection Counter can, however, underestimate the number of infected cells. An example of this highlighted with a white arrow in Figure 2C; visual inspection suggests a group of four infected cells whereas Infection Counter identifies two infected cells. Therefore, to test whether Infection Counter was capable of achieving comparable levels of accuracy to manual counting in a FFA, we performed three independent serial dilutions of HCVcc on Huh-7.5 cells, determined the titer by standard FFA and analyzed images of replicate wells using Infection Counter (Figure 3). Infection Counter reliably quantified HCV titers ranging from ~300 to >9000 FFU/mL, indicating a linear dynamic range of at least thirty-fold, which is comparable to other automated detection method [1,2,3]. Notably, there was a near perfect linear relationship between the automated and manual quantification methods (linear regression; slope = 1.046 ± 0.02 when normalized for differences in units); this suggests that a standard calibration plot could be used to interpolate conventional viral titers from Infection Counter data.

**Figure 4:**
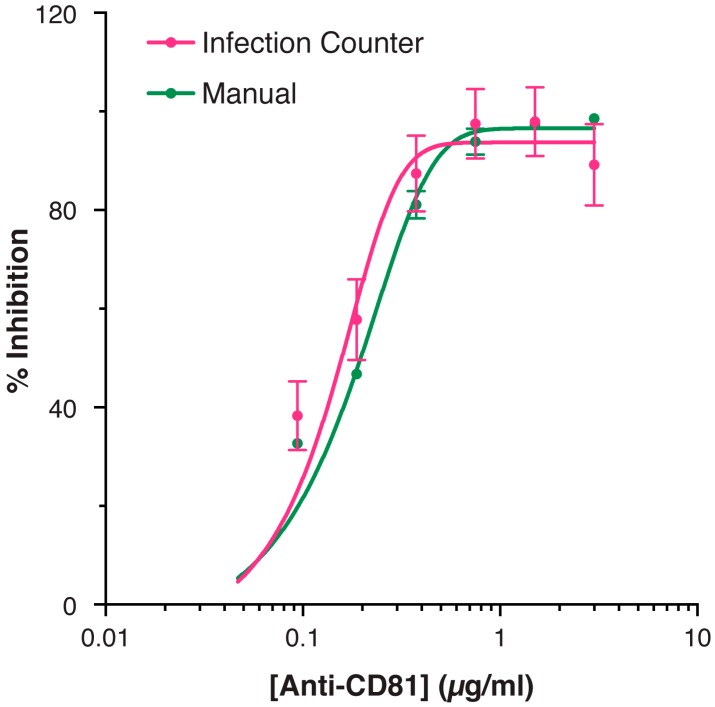
HCV receptor blockade by anti-CD81 monoclonal antibody (mAb). Huh-7.5 cells were pretreated for 1 h at 37 °C with a serial dilution of anti-CD81 mAb 2.20, after which the cells were inoculated with J6/JFH HCVcc in replicates of four. The cells were fixed after 48 h, stained for viral antigen and nuclear DNA, and imaged using a plate-reading fluorescence microscope. The infection in each well was quantified using the standard FFA (‘Manual’) and the proportion of infected cells was estimated using Infection Counter. The data is expressed as percentage of inhibition relative to Huh-7.5 cells treated with an irrelevant control mAb. Sigmoidal curves were fitted using non-linear regression (*R*^2^ = 0.91 for both curves) and F-test comparison indicated no statistically significant difference between the manual and Infection Counter data (*p* = 0.29). Error bars indicate standard deviation from the mean, *n* = 2.

To further validate the method we assessed the inhibitory activity of HCV receptor blockade by an anti-CD81 mAb [10]. Commensurate with the essential role for CD81 in virus entry [11], mAb treatment potently inhibited HCV infection with an IC_50_ of ~0.2 μg/mL (1.3 nM) (Figure 4). Quantification by standard FFA or Infection Counter analysis yielded statistically indistinguishable dose response curves (F-test, *p* = 0.29). This confirms that Infection Counter produces data that are equivalent to standard manual quantification.

### 3.3. Implementation

**Figure 5:**
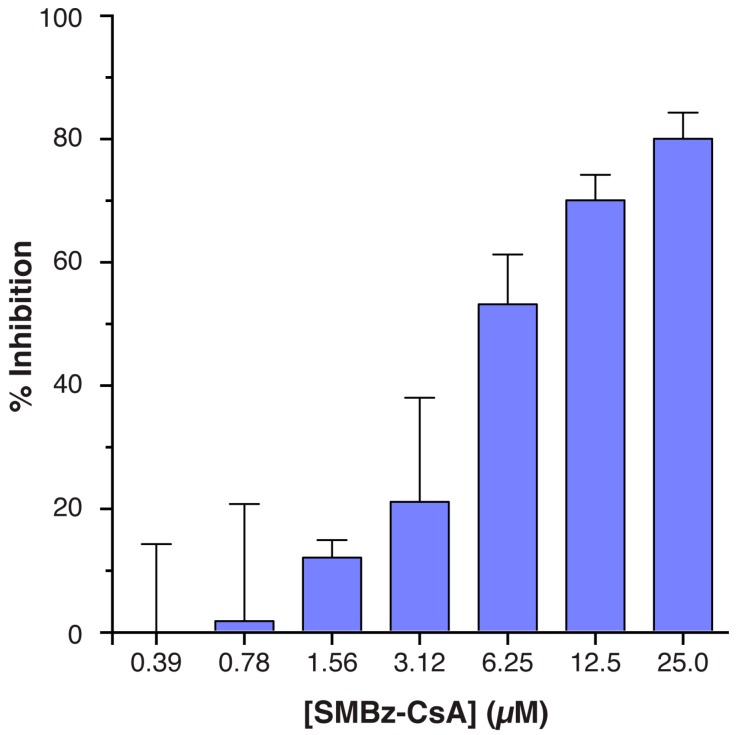
The non-immunosuppressive cyclosporine analogue SMBz-CsA inhibits HCVcc replication. Huh-7.5 cells were pretreated for 1 h at 37 °C with a serial dilution of SMBz-CsA, after which the cells were inoculated with J6/JFH HCVcc in duplicate. After 6 h the inoculum was removed and the cells re-fed with media without drug. The samples were fixed after 48 h, stained for viral antigen and nuclear DNA, and imaged using a plate reading microscope. The infection was then quantified using Infection Counter. The data is expressed as percentage of inhibition relative to Huh-7.5 cells treated with dimethyl sulfoxide (DMSO) control. Error bars indicate standard deviation from the mean, *n* = 3.

Automated quantification of viral replication provides a medium/high-throughput assay for basic and translational research. We exploited this to assess the ability of a cyclosporine (CsA) analogue, SMBz-CsA [12,13,14], to inhibit the HCV life cycle. CsA is an immunosuppressive drug. When in complex with its target, cyclophilin A (CypA), it blocks T-cell activation by inhibition of the phosphatase calcineurin [15]. CypA is an important cellular co-factor for HCV; it is thought to be required for proper assembly of the HCV replication complex, likely through interactions with NS5A [16,17,18,19,20,21,22,23]. CsA potently inhibits HCV replication in vitro, this is largely attributed to its ability to block CypA-NS5A interactions, which disrupts the formation of the double membrane vesicles necessary for HCV replication [16,17,18,19,20,21,22,23]. Moreover, in vivo data demonstrate that the non-immunosuppressive CsA analogue SCY635 enhances interferon-α/β production in HCV-infected individuals [21]. A further study in chronically infected patients demonstrated that the non-immunosuppressive CsA analogue Debio 025 enhanced the anti-HCV activity of pegylated interferon-alpha (PEG IFN-α) [24]. We used Infection Counter to assess the inhibitory activity of SMBz-CsA, an alternative non-immunosuppressive CsA analogue. Huh-7.5 cells were treated with SMBz-CsA before and during a 6 h inoculation with J6/JFH HCVcc. Importantly, we observed no evidence of cytotoxicity upon treatment with SMBz-CsA, as assessed by cell density in each well (data not shown). Virus replication was assessed after 48 h using Infection Counter (Figure 5). Consistent with previous reports, SMBz-CsA exhibited robust inhibition of HCV replication with an IC_50_ of ~7 μM. In this experiment, the drug was only present during early infection (0–6 h), prior to the later events of genome replication that are thought to be the principal targets of CsA-mediated inhibition of HCV. This may suggest that SMBz-CsA possesses inhibitory activity against earlier stages of the HCV life cycle such as entry, un-coating or initial translation. Alternatively, SMBz-CsA may possess sufficient intracellular stability to block the late stages of replication. A full appreciation of when in the HCV life cycle SMBz-CsA is active will require detailed time of addition studies.

## 4. Discussion

In this report we describe the development, validation and implementation of Infection Counter, a robust method for automated quantification of in vitro HCV replication. Other groups have reported similar techniques [1,2,3], however these have generally required bespoke equipment and/or commercial software. In contrast, Infection Counter is freely available as an open-source plugin for ImageJ and Fiji analysis software, and benefits from being relatively simple (for example, there are only four user-dependent parameters). Whilst the data generated for this report were collected on a plate-reading microscope, any fluorescence microscope would suffice; therefore, we suggest that this method is a more accessible alternative to other approaches.

We found Infection Counter to be a robust method for measuring viral titer when tested against a serial dilution of HCVcc; the estimate percentage of infected cells displayed a near perfect linear relationship to absolute virus concentration (as measured using manual FFA) over a dynamic range of at least thirty-fold (Figure 3). We also compared manual FFA to Infection Counter for calculating the inhibitory activity of HCV receptor-blockade by anti-CD81 mAb (Figure 4). The resultant dose response curves were statistically indistinguishable, indicating that measurements obtained using Infection Counter are equivalent to the standard FFA. Importantly, Infection Counter is more efficient and less prone to operator error than manual methods, allowing an increase in throughput of viral infectivity assays. For instance, using an appropriate plate reading microscope and Infection Counter, a 96-well plate can be imaged and quantified in ~1 h, performing the same measurements by standard FFA would require many hours of analysis by a dedicated operator.

Alternative methods for medium/high-throughput analysis of viral infection are available. For instance, viruses encoding luciferase reporters are commonly used in HCV research [25,26,27]. This method is arguably quicker than a fluorescence based approach, as there are fewer sample processing steps and the read out is very rapid. However, not all viruses can accommodate a genetically encoded reporter. Furthermore, unlike microscopy-based methods, luciferase assays generally require cell lysis and therefore cannot assess the proportion of infected cells or how they are distributed in the monolayer. This can be useful information; for instance, a hallmark of HCV replication is the appearance of distinct foci, containing numerous infected cells (Figure 1 and Figure 2). The size and distribution of these foci reflects the relative contributions of cell-free and cell-to-cell transmission [28]. Infection Counter can provide the point co-ordinates of virus positive cells (see Supplementary Note 1, optimisation mode), such data is amenable to spatial statistics analysis [29,30] to better quantify virus transmission. Whilst this type of analysis is not currently integrated in to Infection Counter, future releases may include these and other features.

Finally, we implemented Infection Counter to assess the inhibitory activity of SMBz-CsA, a non-immunosuppressive CsA analogue. Consistent with other reports, SMBz-CsA inhibited HCVcc replication (Figure 5). This is likely to have occurred through perturbation of CypA-NS5A interactions. However, given the apparent activity of the drug during early infection other mechanisms may also be at play. CypA has recently been shown to orchestrate the evasion of intracellular innate immune responses by human immunodeficiency virus (HIV) [14]. Whether HCV commandeers CypA to a similar aim remains to be investigated, although it should be noted that CsA has been previously shown to enhance interferon production in HCVcc infected cells [21,31]. SMBz-CsA exhibits inhibitory activity against distinct viral pathogens including HCV and HIV, given this, its use as a pan-anti-viral warrants further study.

The automated analysis method described here is available as an ImageJ plugin. It is free to be improved, adapted or appropriated, and we urge readers to try it out on their virus of choice.

## Supplementary Materials

The following are available online at www.mdpi.com/link/1999-4915/8/7/201/s1, Supplementary Note 1 (detailed instructions on how to use the plugin), Supplementary Data 1 (an example image for analysis), Supplementary Software (InfectionCounter.jar file for use with ImageJ/FIJI).
