# Supplementary material for: Infection Counter: Automated Quantification of in Vitro Virus Replication by Fluorescence Microscopy
Source: Viruses. 2016 Jul 21;8(7):201. doi: 10.3390/v8070201 (PMC4974536; doi:10.3390/v8070201)
Supplement: Supplementary file 1 [file viruses-08-00201-s001.zip › viruses-129433-supplementary/Supplementary Note 1.docx]

Supplementary Note 1: How to use the Infection Counter ImageJ plugin.

**­1. Overview**

The Infection Counter plugin estimates the percentage of virus positive cells in images of infected cell monolayers. The plugin batch processes two channel tif files (in a chosen folder) where channel 1 displays DAPI-stained cell nuclei and channel 2 (the target channel) shows viral antigen labeled by immunofluorescence. It uses a simple method to segment the cells based on their nuclei and then measures the fluorescence signal associated with each cell in the target channel. Virus positive cells are then scored based on an empirically chosen signal threshold. This document provides a step-by-step guide to using the plugin and some additional advice for successful quantification. For readers interested in the technical details of image analysis, the ImageJ macro (upon which the Infection Counter plugin is based) is provided in Appendix A.

2. Using the plugin

*2.1 Installation.*

The plugin can be installed in two ways. The first method is to copy the latest InfectionCounter_.jar file (provided in the supplementary material and available at <https://github.com/jgrovelab/InfectionCounter/releases>) into the Plugins folder of the Fiji/ImageJ application. Fiji/ImageJ should be restarted after the .jar file has been added to this folder. The second method is to add the Infection Counter update site to Fiji by choosing ‘Update Fiji’ from the ‘Help’ menu. This will open the ‘ImageJ Updater’ window; press the ‘Manage update sites’ button to open the ‘Manage update sites’ window. In ‘Manage update sites’, press ‘Add my site’, and then type InfectionCounter into the ‘ImageJ Wiki Account’ field and press OK. Close the ‘Manage update sites window’ and press ‘Apply changes’ in the ‘ImageJ Updater’ window. Fiji will then need to be restarted.

*2.2 Running the plugin.*

Following installation as above and restarting Fiji/ImageJ, Infection Counter can be run from the ‘Quantitative Viral Imaging’ menu option in the Plugins menu (Figure S1).


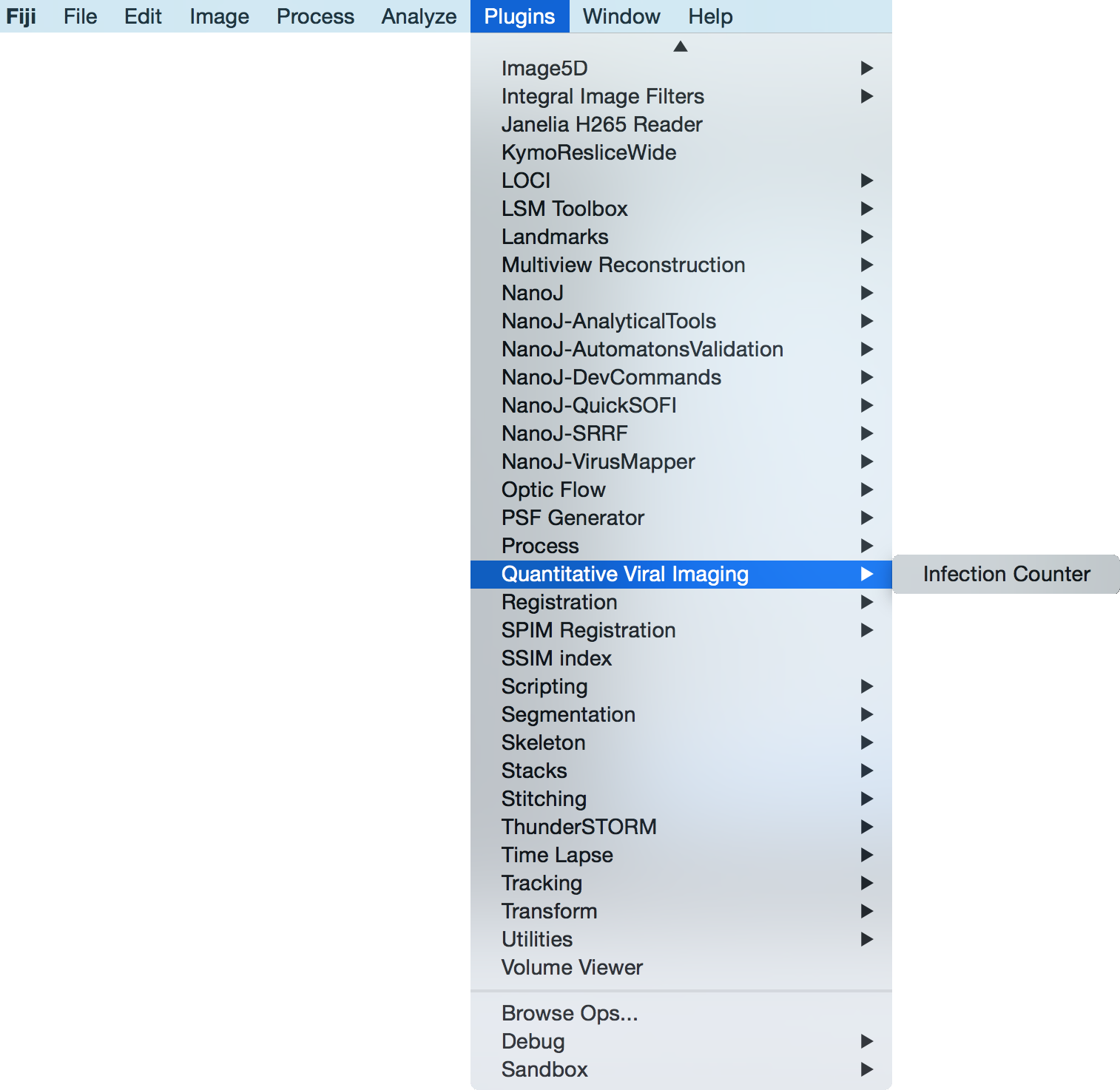


**­Figure S1.** Accessing Infection Counter from the Fiji/ImageJ plugins menu.

*2.3 User interface.*

Infection Counter has four critical parameters that need to be optimised to individual experimental procedures. Running the plugin will launch a user interface for inputting appropriate values (Figure S2). Once optimised these parameters are robust and should work consistently for a standardised assay.


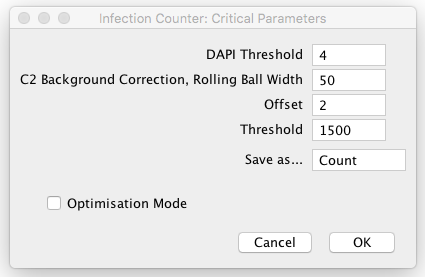
**Figure S2** User Interface.

*2.3.1 DAPI threshold:* Infection Counter uses the ‘Find Maxima’ function to identify individual DAPI-stained nuclei, more can be found on this function at http://rsbweb.nih.gov/ij/docs/guide/146-29.html. The inputted value determines the amount by which local maxima must stand out from the surrounding noise. Once set appropriately this will correctly identify the vast majority of nuclei.

*2.3.2 C2 Background Correction, Rolling Ball Width:* Infection Counter uses the ‘Rolling Ball Background Subtraction’ function to remove noise from the target channel, more on this function can be found at http://fiji.sc/Rolling_Ball_Background_Subtraction. This value determines the radius of the rolling ball (in pixels) and should be set to at least the size of the largest structures you are intending to measure (e.g. a cell body). This will remove much of the background signal and should mitigate against uneven illumination across an image.

*2.3.3 Offset:* This value is subtracted from each pixel in the image, which can be useful for eliminating residual background; maximizing the signal to noise ratio will greatly improve the performance of Infection Counter.

*2.3.4 Threshold:* Infection Counter measures the sum total of fluorescence signal associated with a segmented cell. The inputted value determines the minimum signal intensity value required for a cell to be scored as positive. This should be robust within a range of values but will need to be empirically chosen.

*2.3.5 Save as:* The data will be saved in to the target folder using this file name.

*2.3.6 Optimisation Mode:* This allows plugin performance to be reviewed and is particularly useful when optimising the critical parameters. Selecting optimisation mode will instruct the plugin to save summary files in the target folder. Three images are generated, which, when opened in ImageJ, will be annotated as follows: ‘_C1_processed’ displays the DAPI channel with the identified nuclei highlighted as a point selection, ‘_C2_processed’ displays the background subtracted target channel with positive cells highlighted as a point selection, and ‘_voronoi’ displays the Voronoi mosaic used to approximate cell bodies with the accompanying regions of interest as an overlay. Example images can be seen in Figure S3. The ‘ _data’ file is a Microsoft Excel compatible table displaying the x y coordinates of each approximated cell body and their associated signal intensities, this can be useful when choosing an appropriate threshold value for identifying positive cells.


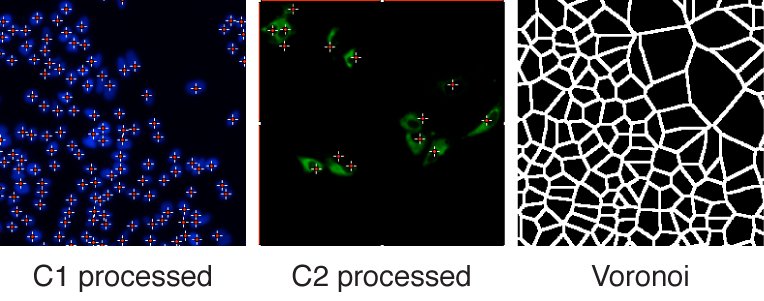


**Figure S3** Example of images generated during optimisation mode.

*2.4 Batch processing.*

The plugin batch processes images in a target folder (not the images currently open in ImageJ). After clicking ‘OK’ the plugin will request a target folder and work through each two channel .tif file by alphabetical order. When initially optimising the parameters within Infection Counter it is recommended that you select a folder containing a small number of representative images.

*2.5 Data output.*

The data is saved, in the target folder, as a Microsoft Excel compatible .csv file containing a list of percent infection for each image, followed by a record of the parameters used to analyse the data set.

An example image of HCVcc infected cells is provided in Supplementary Data 1. This can be used to familiarise yourself with Infection Counter and test critical parameters before using the plugin with your own data. The default values in the user interface should work well with this image.

3. Other considerations

Infection Counter provides a robust and reliable means of quantifying virus-infected cells. Moreover, it could be easily adapted to any situation where the proportion of fluorescent cells needs to be estimated. There are a number of practical considerations when applying Infection Counter to images.

*3.1 Sample preparation.*

The background subtraction and offset functions within Infection Counter will remove noise from images. However, there is no substitute for a good antibody and a standardised staining protocol. Consider optimising each step of the procedure: suppress fixation-associated autofluorescence with quenching; titrate the primary and secondary antibodies; spin out aggregated antibody before preparing staining buffers; where appropriate, test detergents to reduce non-specific binding. Also note that Infection Counter does not perform particularly well at very low cell densities.

*3.2 Assay range and linearity.*

When testing this or similar approaches perform a titration of virus (Figure 3 of the main text) to evaluate the range and linearity of the assay and provide confidence in the methodology. Note that the intensity of staining is an important determinant of assay range; insufficient primary or secondary antibody will impair the ability of Infection Counter to accurately quantify high proportions of infected cells.

*3.3 Data acquisition.*

Increasing the size and number of images will improve the sensitivity of detection. Also consider the quality and sensitivity of the camera being used.

Appendix A Infection Counter macro script.

// Infection Counter, Joe Grove, 2016

// This ImageJ macro was designed to estimate the percentage of virally infected cells in a monoloayer

// based on the intensity of immunofluorescently labeled viral antigen.

// An up to date version of the code is maintained at https://github.com/jgrovelab/InfectionCounter

// This text is distributed under a creative commons Attribution-NonCommercial license

// http://creativecommons.org/licenses/by-nc/3.0/

// Feel free to adapt, improve or appropriate this script to suit your needs.

// The author offers no guarantees of the macro's performance.

// However, it has been tested on multiple Windows and OSX based machines.

// This macro processes two channel tif stacks where channel 1 displays cell nuclei and channel 2 (the target channel)

// displays viral antigen.

// Script starts. /////////////////////////////////////////////////////////////////////////////////////////////////////

// A. User interface. /////////////////////////////////////////////////////////////////////////////////////////////////

// This allows input of critical parameters.

// These will need to be optimised to fit individual experimental procedures.

// Recall preferences

DAPI = call("ij.Prefs.get", "InfectionCounter.DAPI", "4");

Background = call("ij.Prefs.get", "InfectionCounter.Background", "50");

Offset = call("ij.Prefs.get", "InfectionCounter.Offset", "2");

Threshold = call("ij.Prefs.get", "InfectionCounter.Threshold", "1500");

SaveName = call("ij.Prefs.get", "InfectionCounter.SaveName", "Count");

// Create dialog

Dialog.create("Infection Counter: Critical Parameters");

// The noise threshold to find DAPI maxima.

Dialog.addNumber("DAPI Threshold", DAPI);

// The rolling balling width for background correction of the target channel (channel 2).

Dialog.addNumber("C2 Background Correction, Rolling Ball Width", Background);

// A value to be deducted from the target channel (channel 2). This can be used to remove all non-specific signal.

Dialog.addNumber("Offset", Offset);

// The threshold value for a cell to be scored as positive in the target channel (channel 2).

Dialog.addNumber("Threshold", Threshold);

// Save data as

Dialog.addString("Save as...", SaveName);

// Optimisation mode saves processed and annotated images and a data file. This is useful when optimising parameters.

Dialog.addCheckbox("Optimisation Mode", false);

Dialog.show();

// Gather values from interface.

DAPI=Dialog.getNumber();

Background=Dialog.getNumber();

Offset=Dialog.getNumber();

Threshold=Dialog.getNumber();

SaveName= Dialog.getString()

Optimisation= Dialog.getCheckbox();

// Write and save preferences

call("ij.Prefs.set", "InfectionCounter.DAPI", DAPI);

call("ij.Prefs.set", "InfectionCounter.Background", Background);

call("ij.Prefs.set", "InfectionCounter.Offset", Offset);

call("ij.Prefs.set", "InfectionCounter.Threshold", Threshold);

call("ij.Prefs.set", "InfectionCounter.SaveName", SaveName);

call("ij.Prefs.savePreferences");

// B. Batch analysis. /////////////////////////////////////////////////////////////////////////////////////////////////

// Clear ROI manager and log.

roiManager("Reset");

print("\\Clear");

// Initiate batch analysis.

requires("1.33s");

dir = getDirectory("Choose a Directory");

setBatchMode(true);

count = 0;

countFiles(dir);

n = 0;

processFiles(dir);

function countFiles(dir) {

list = getFileList(dir);

for (i=0; i<list.length; i++) {

if (endsWith(list[i], "/"))

countFiles(""+dir+list[i]);

else

count++;

}

}

function processFiles(dir) {

list = getFileList(dir);

for (i=0; i<list.length; i++) {

if (endsWith(list[i], "/"))

processFiles(""+dir+list[i]);

else {

showProgress(n++, count);

path = dir+list[i];

processFile(path);

}

}

}

function processFile(path) {

if (endsWith(path, ".tif")) {

open(path);

// C. Analysis Pipeline. /////////////////////////////////////////////////////////////////////////////////////////////////

// 1. Ensure image has the correct channel dimensions.

run("Stack to Hyperstack...", "order=xyczt(default) channels=2 slices=1 frames=1 display=Color");

// 2. Get image name.

name=getTitle;

// 3. Get image dimensions.

W=getWidth();

H=getHeight();

// 4. Remove scale information.

run("Set Scale...", "distance=1");

// 5. Trim edges by 5 pixels - this eliminates a potential source of artifacts.

makeRectangle(5, 5, (W-10), (H-10));

run("Crop");

// 6. Split channels.

selectWindow(name);

run("Split Channels");

// 7. Find nuclei in DAPI channel (channel 1).

selectWindow("C1-"+name);

run("Find Maxima...", "noise=DAPI output=[Point Selection]");

roiManager("Add");

roiManager("Select", 0);

run("Add Selection...");

// 8. Create voronoi mosaic to approximate cell bodies.

setForegroundColor(250, 250, 250);

newImage("Voronoi", "8-bit black", W, H, 1);

selectWindow("Voronoi");

roiManager("Select", 0);

roiManager("Draw");

run("Make Binary");

run("Voronoi");

setThreshold(1, 255);

run("Convert to Mask");

run("Dilate");

// 9. Add voronois to ROI manager.

roiManager("Reset");

selectWindow("Voronoi");

run("Invert LUT");

run("Analyze Particles...", "add");

// 10. Remove background from target channel (channel 2).

selectWindow("C2-"+name);

run("Subtract Background...", "rolling=Background");

// 11. Subtract offset value from target channel (channel 2).

run("Subtract...", "value=Offset stack");

// 12. Measure fluorescence signal density for each ROI in target channel (channel 2).

run("Set Measurements...", "centroid integrated redirect=None decimal=0");

nROI = roiManager("count");

for (n = 0; n < nROI; n++){

roiManager("select", n);

roiManager("Measure");

}

// 13. Calculate percentage positive cells, based on chosen threshold, and add to log.

counter = 0;

for (n=0; n<nResults; n++) {

Signal = getResult("IntDen", n);

if (Signal > Threshold) {

counter++;

}

}

print(100*counter/nResults);

// 14. Optimisation mode.

// This saves summary images for optimisation.

if (Optimisation){

// 14A. Annotate positive cells on target channel (channel 2).

roiManager("Reset");

selectWindow("C2-"+name);

for (n=0; n<nResults; n++) {

intDen = getResult("IntDen", n);

x = getResult("X", n);

y = getResult("Y", n);

if (intDen > Threshold) {

makePoint(x, y);

roiManager("Add");

run("From ROI Manager");

}

}

// 14B. Save annotated images and data file.

saveAs("Results", path+"_data.csv");

selectWindow("C1-"+name);

saveAs("Tiff", path+"_C1_processed");

selectWindow("C2-"+name);

saveAs("Tiff", path+"_C2_processed");

selectWindow("Voronoi");

saveAs("Tiff", path+"_voronoi");

}

// Optimisation mode ends.

// 15. Clear up prior to next loop

roiManager("Reset");

run("Clear Results");

run("Close All");

}

}

// 16. Add analysis parameters to log and save data

print("Parameters")

print("DAPI Threshold")

print(DAPI);

print("Background Correction");

print(Background);

print("Offset");

print(Offset);

print("Threshold");

print(Threshold);

selectWindow("Log");

saveAs("text", dir+SaveName+".csv");

// Script end. /////////////////////////////////////////////////////////////////////////////////////////////////////////////
