# Supplementary figures and images for: Infection Counter: Automated Quantification of in Vitro Virus Replication by Fluorescence Microscopy
Source: Viruses. 2016 Jul 21;8(7):201. doi: 10.3390/v8070201 (PMC4974536; doi:10.3390/v8070201)

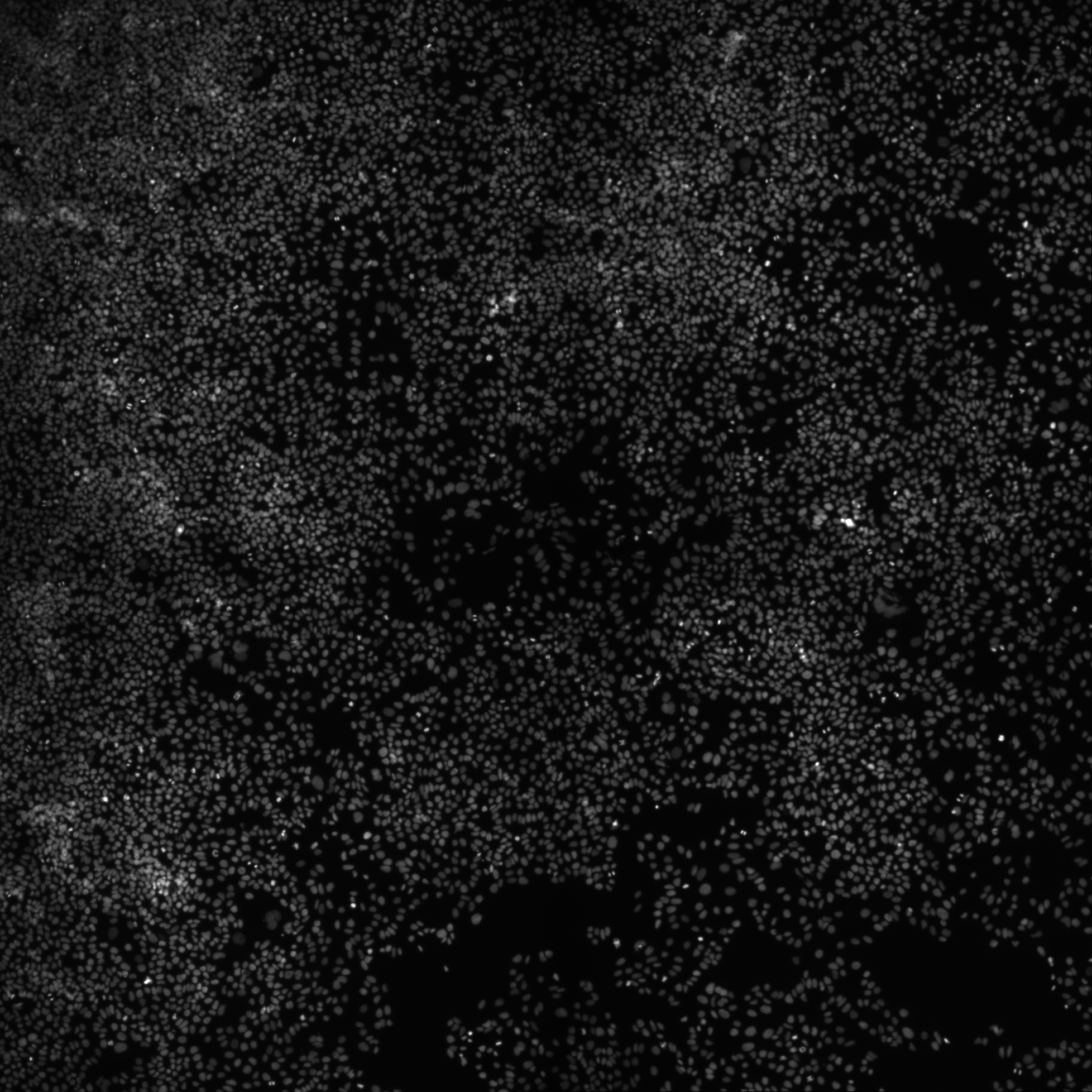

Supplement: Supplementary file 1 [file viruses-08-00201-s001.zip › viruses-129433-supplementary/Supplementary Data 1 Example Image.tif]
